# Early childhood development strategy for the world’s children with disabilities

**DOI:** 10.3389/fpubh.2024.1390107

**Published:** 2024-06-19

**Authors:** Bolajoko O. Olusanya, Scott M. Wright, Tracey Smythe, Mary A. Khetani, Marisol Moreno-Angarita, Sheffali Gulati, Sally A. Brinkman, Nihad A. Almasri, Marta Figueiredo, Lidia B. Giudici, Oluwatosin Olorunmoteni, Paul Lynch, Brad Berman, Andrew N. Williams, Jacob O. Olusanya, Donald Wertlieb, Adrian C. Davis, Mijna Hadders-Algra, Melissa J. Gladstone

**Affiliations:** ^1^Centre for Healthy Start Initiative, Lagos, Nigeria; ^2^Johns Hopkins Medicine, Johns Hopkins University, Baltimore, MD, United States; ^3^London School of Hygiene and Tropical Medicine, University of London, London, United Kingdom; ^4^Department of Occupational Therapy, College of Applied Health Sciences of Illinois Chicago, Chicago, IL, United States; ^5^Department of Disability Studies, the National University of Colombia, Bogotá, Colombia; ^6^All India Institute of Medical Sciences, New Delhi, India; ^7^Fraser Mustard Centre, Telethon Kids Institute, School of Public Health, The University of Adelaide, Adelaide, SA, Australia; ^8^School of Rehabilitation Sciences, The University of Jordan, Amman, Jordan; ^9^European Network of Occupational Therapy in Higher Education, Escola Superior de Saúde do Alcoitão, Alcabideche, Portugal; ^10^Latín American Association for Neonatal and Pediatric Follow-Up (ALSEPNEO), Buenos Aires, Argentina; ^11^College of Health Sciences, Obafemi Awolowo University, Ife, Nigeria; ^12^School of Education, University of Glasgow, Glasgow, United Kingdom; ^13^Benioff Children’s Hospital, University of California, San Francisco, San Francisco, CA, United States; ^14^Virtual Academic Unit, Children’s Directorate, Northampton General Hospital, Northampton, United Kingdom; ^15^Eliot-Pearson Department of Child Development, Tufts University, Medford, MA, United States; ^16^Anglia Ruskin University, Cambridge, United Kingdom; ^17^University Medical Centre Groningen, Department of Pediatrics, Division of Developmental Neurology, and Faculty of Theology and Religious Studies, University of Groningen, Groningen, Netherlands; ^18^Department of Women's and Children's Health, Institute of Translational Medicine, University of Liverpool, Liverpool, United Kingdom

**Keywords:** developmental disabilities, early childhood development, global strategy, school readiness, inclusive education, nurturing care framework, Sustainable Development Goals, twin track approach

## Abstract

Early childhood is foundational for optimal and inclusive lifelong learning, health and well-being. Young children with disabilities face substantial risks of sub-optimal early childhood development (ECD), requiring targeted support to ensure equitable access to lifelong learning opportunities, especially in low- and middle-income countries. Although the Sustainable Development Goals, 2015–2030 (SDGs) emphasise inclusive education for children under 5 years with disabilities, there is no global strategy for achieving this goal since the launch of the SDGs. This paper explores a global ECD framework for children with disabilities based on a review of national ECD programmes from different world regions and relevant global ECD reports published since 2015. Available evidence suggests that any ECD strategy for young children with disabilities should consists of a twin-track approach, strong legislative support, guidelines for early intervention, family involvement, designated coordinating agencies, performance indicators, workforce recruitment and training, as well as explicit funding mechanisms and monitoring systems. This approach reinforces parental rights and liberty to choose appropriate support pathway for their children. We conclude that without a global disability-focussed ECD strategy that incorporates these key features under a dedicated global leadership, the SDGs vision and commitment for the world’s children with disabilities are unlikely to be realised.

## 1 Introduction

Early childhood development (ECD) is foundational for optimal learning, health and wellbeing over the life-course and a nation’s human capital development (1). This recognition is reflected in global agendas like the Incheon Declaration on Education 2030 (2), and the Sustainable Development Goals, 2015–2030, (SDGs) (3). The SDGs include a specific target to ensure that by 2030 children under 5 years of age (defined as “children under-5” hereinafter) can access quality ECD in readiness for primary education (SDG 4.2) (3). Globally, over 50 million children under-5 have mild-to-severe disabilities predominantly in low- and middle-income countries (LMICs), with 30 million having moderate-to-severe disabilities (4, 5). Childhood disabilities are diverse in nature, type and severity and are associated with functional difficulties typically from hearing impairment, visual impairment, deaf-blindness, speech and language disorders, intellectual disability, learning disabilities, autism spectrum disorder, attention-deficit/hyperactivity disorder, epilepsy, cerebral palsy, muscular dystrophies, spina bifida or multiple disorders that require wide-ranging support services (6). The disproportionate disadvantages faced by children with disabilities compared to children without disabilities, including higher risk of morbidity, premature death, lower rates of school enrolment and completion, and social exclusion are widely reported (4, 7, 8). Also recognised is the substantial emotional, health, psychosocial and economic impact of childhood disability on the affected families (9–11), and the need to prioritise children with disabilities in any global ECD initiatives (4, 12). However, there is presently no strategy to implement the global ECD agenda towards school readiness for children under-5 with disabilities, especially in LMICs (13–15). Such a strategy is needed to provide a unifying framework and action plan among UN member states and international developmental assistance providers for the effective implementation of global commitment on ECD (16–18). The only existing global ECD initiative - the “Nurturing Care Framework” (NCF) – focuses on the first 1,000 days from conception (19) and was not designed to promote school readiness for children under-5 with and without disabilities (15). In this paper, we discuss the need and features of an appropriate global disability-focussed ECD strategy for children under-5 with disabilities based on an overview of well-established national ECD programmes in different world regions. ECD policies and programmes designed to serve all children from birth to school entry (5–6 years) are termed “disability-inclusive,” while those designed exclusively to identify and support children with developmental delays and disabilities are termed “disability-focused” or “disability-specific.”

## 2 Data sources

A global survey of ECD programmes published in 2019 by the Early Childhood Development Task Force in collaboration with UNICEF reported 426 programmes from 121 countries (20). The largest number of programmes were reported from Sub-Saharan Africa (*n* = 115 or 27%), and the least number from the Middle East and North Africa (*n* = 14 or 3.3%). To identify relevant national ECD programmes from different world regions we examined ECD and inclusive education reports published after the launch of the SDGs by UNICEF, WHO, the World Bank, UNESCO, and the Organisation for Economic Cooperation and Development (OECD) and USAID (21–26). We also reviewed global disability-related ECD reports published between 2015 and 2023 by International Disability and Development Consortium, International Disability Alliance, and major funders of disability projects in LMICs, including USAID and DFID to complement the findings from the national ECD programmes (Appendix 1).

After an interactive session on the primary goal of this review and prior publications by GRDDC, we chose 10 key criteria for selecting ECD programmes for our analysis namely: the existence of a national policy or programme, date of establishment of at least 10 years, relevant legislations, target beneficiaries, type of disability services offered, designated service providers, performance indicators, budget or disbursements, governance structure and open data sources. We were unable to select countries based on indicators such as the rate of school enrolment or drop-out rate among children with disabilities because of the general lack of publicly available population-based data particularly in LMICs (4, 5).

## 3 Overview of national ECD programmes

Fifteen national ECD programmes from 11 countries (three high-income countries or HICs and eight LMICs countries) were purposively selected (27), based on sufficient publicly available information on the parameters listed in Table 1, including countries with substantial prevalence of children with disabilities (4, 28). Three countries (Kenya, Nigeria and South Africa) were selected from sub-Saharan Africa, three (Brazil, Chile and Jamaica) from Latin America and the Caribbean, and two countries (Bangladesh and India) from South Asia. Additionally, one HIC each was included from North America (United States of America), Europe (United Kingdom), and East Asia/Pacific (Australia). Limited data were available from Middle-East and North Africa on disability-inclusive or disability-focused ECD as per the chosen criteria (25, 26). The 11 countries selected account for approximately 39% of the estimated 3 million children with disabilities in HICs and 35% of the 50 million children with disabilities in LMICs (28).

**Table 1:** Overview of selected national early childhood development programmes for children with disabilities.

	High-income countries							Low- and middle-income countries							
Country	Australia	Australia	United Kingdom	United Kingdom	United States of America	United States of America	United States of America	Bangladesh	Brazil	Chile*	India	Jamaica	Kenya	Nigeria	South Africa
Programme or Policy	Early Childhood Targeted Action Plan in the Australia’s Disability Strategy 2021–2031	The NDIS Early Childhood Early Intervention (ECEI)	Early Years Foundation Stage (EYFS)	Children with Special Educational Needs and Disabilities (SEND)	Early Head Start & Head Start	IDEA Part C Early Intervention Programme and Part B Preschool Education Programme	Preschool Development Grant Birth to Five (PDG B to 5)	The Comprehensive Policy for Early Childhood Care and Development	Special Education Guidelines for Early Childhood Education	Chile Crece Contigo (ChCC, Chile Grows with You)	Rashriya Bal Swasthya Karyakram (RBSK)	National Strategic Plan for Early Childhood Development 2008–2023	The National Early Childhood Development Policy Framework	The National Policy for Integrated Early Childhood Development (IECD) in Nigeria.	The National Integrated Early Childhood Development Policy
Date Established	2010, National Disability Strategy 2010–2020.	2013	2006, Childcare Act	1989 as Children Act, Updated in 2002	1965	1975 [as: Education for All Handicapped Children Act (EAHCA), renamed IDEA in 1990]	2002 [as: No Child Left Behind (NCLB) Act]	2013	2015 [Previously as National Policy on Special Education 1994]	2007	2013	2003	2006	2007	2005, Children’s Act No. 38
Current Legislation	Disability Discrimination Act, 1992, National Standards For Disability Services (NSDS), 2014, National Disability Insurance Scheme Act, 2013	NDIS Act (National Disability Insurance Scheme Act). 2013. No. 20. Canberra	Early Years Foundation Stage (EYFS) Statutory Framework 2014, updated 2021	Children and Families Act 2014 (Part 3)	Improving Head Start for School Readiness Act of 2007	Individuals with Disabilities Education Act, 2004	Every Student Succeeds Act (ESSA), 2015	The Children Act 2013 (“Shishu Ain, 2013”)	The Legal Framework for Early Childhood, Law No. 13,257, OF MARCH 8, 2016; amends Law No. 8069, of July 13, 1990 (Statute of the Child and Adolescent).	Chile National Law 20,379 of 2009	Government of India Executive Orders, 2013	Early Childhood Commission Act, 2003 & Early Childhood Act and Regulations, 2005, The Disabilities Regulations 2021	Early Childhood Education Act, 2021	Education Reform Act, 2007	Cabinet Approval in 2015. National ECD legislation under consideration.
Target beneficiaries	Children 0–6 years with disabilities	All children 0–9 years including those with disabilities	All children 0–5 years including those with disabilities	Children 0–25 years with special educational needs and disabilities	All children in low-income families, homeless families, families receiving public assistance and foster children. Poverty guideliness are issued by the Department of Health and Human Services.	Children with disabilities, including infants, toddlers, and youth based on a list of 13 disability categories, including optional “developmental delay” category	Vulnerable, disadvantaged, and underserved children under 5 years including children with disabilities	All children from conception to age 8 years including children with disabilities	All children 0–6 years including those with disabilities	All Children from birth to age 9 years including those with a disability and belonging to households in the lower 60% of the national income distribution according to the Ministry of Social Development’s socioeconomic classification.	All children below 18 years with defects at birth, development delays/disabilities, deficiencies, and specified diseases	All children 0–6 years including those with disabilities	All children from conception to age 8 years including children with disabilities	All children 0–5 years including those with disabilities	All children from birth till a year before formal school entry
Disability Services	Early detection and intervention services, Local capacity building to support parents and caregivers	Counselling, developmental assessment, support services, or referrals, and access to NDIS grants	Education and care of all children in early years, including children with special educational needs and disabilities (SEND).	Developmental Assessment at age 2 years, Health Check by Health Visitor at age 2–3 years	Educational, nutritional, health, social, and other essential services	Early Detection and Intervention Services in child’s natural environment, as outlined in an Individualised Family Service Plan (Part C), and Free Appropriate Public Education in least restrictive environment as outlined in an Individualised Education Program (Part B). Child Find Systems to locate, identify, and evaluate children for eligibility.	Strengthening early childhood development, care, and education system; improving screenings, referrals, and support for children with disabilities	Ensure early detection of disability and special needs for appropriate interventions.	Provision early detection and management of children with disabilities	Free techincal assistance for children with disabilities by the provision of assistive technologies.	Community-based and facility-based early detection and intervention	Early and effective screening, diagnosis and early intervention for at-risk children and households	Ensure early identification, assessment and interventions of children with special needs and disabilities	Provision early detection and management of children with disabilities	Birth screening and follow-up screening for the early identification of disabilities.
Service Providers	Australian, state, territory and local governments, along with businesses, the community and the non-government sector	Local organisations accredited as early childhood intervention (ECI) partners	Health Visitors & Early Years Providers (Nurseries, Playgroups and Childminders) registered with The Office for Standards in Education (Ofsed)	Health Visitors & Early Years Providers (Nurseries, Playgroups and Childminders) registered with The Office for Standards in Education (Ofsed)	Public agencies, private non-profit and for-profit organisations, tribal governments, and school systems	Public agencies, private non-profit and for-profit organisations, tribal governments, and school systems	State Governments	Health workers, teachers and other ECD providers	Public and private-sector ECD caregivers in Health Centres, Clinics and Pre-School/Day Care facilities.	Identification and referral for specialist services by primary health care providers and community based network of Chile Crece Contigo coordinated ECD services (early education and social protection)	District Early Intervention Centres (DEICs) & Mobile intervention units for the diagnosis, development therapy and physiotherapy.	Accredited Early Childhood Institutions	Approved Early Childhood Development and Education (ECDE) Centres	Public and private-sector ECD caregivers in Health Centres, Clinics and Pre- School/Day Care facilities.	Health Professionals, Educationists, and ECD practitioners accredited by the Department of Social Development
Disability-related Performance Indicators	Participation rate for children with disability 0–6 years in child care services	Number of children 0–6 years with disabilities supported by NDIS	Number of children served, including those with special needs	Number of children with special educational needs and disabilities (SEND) served	Number and proportion of children with disabilities served under Early Head Start and Head Start	Number of children served under Part B and Part C. For Part C, performance indicators include child and family outcomes. For Part B, indicators include early childhood environments, outcomes, and transition.	Number of jurisdictions receiving grants	Not specified	Not specified	Not specified	Number of eligible children served	Not specified	Children enrolled ECDE services in all counties and number of registered ECDE centres	Not specified	Not specified
Budget and funding	Not specified	$36.7 billion in 2022–23, $41.9 billion projected for 2023–24	£3.8 billion (US$4.8 billion) 2022/23. Proportion disbursed on SEND not reported	£645 million (US$806 million) 2021/22	US$10.7 billion budgeted in 2022 by Federal Government	US$10.8 billion appropriated in 2022 by Federal Government, inclusive of US$410 million for Part B and US$496 million for Part C.	US$315 million in Federal Grants	Not specified	The federal government provides technical and financial assistance to states and municipalities. Funding levels not reported	Federal funding available based on eligibility criteria. US$ 78.8 million allocated in 2018. Latest budget/expenditure details not available.	Appropriations: US$274 million (2019–20), US$275 million (2020–21), US$259 million (2021–22)	Federal funding available based on eligibility criteria. Budget/expenditure details not available.	Ministry of Finance to provide funds to counties for support of children with disabilities	Funding by National, State and Local Governments. Data on actual spending on IECD not available.	Federal funding available based on eligibility criteria. Budget/expenditure details not available.
Governance	The Strategy Advisory Council; Department of Social Services	NDIS Quality and Safeguards Commission, Department of Social Services	Department for Education	Department for Health and Department for Education	Administered by the US Department of Health and Human Services	Office of Special Education Programs (OSEP), Department of Education produces an annual report; federal guidelines and regulations; state identifies lead agency to administer program and provide oversight for local compliance	Co-administered by the Department of Health and Human Resources and the Department of Education	Ministry of Women and Children Affairs	The Ministry of Education (Ministério da Educação, MEC)	Ministerio de Desarrollo Social y Familia, MDS (Ministry of Social Development and Family), in partnership with the Ministry of Health, Ministry of Education and all 345 municipalities in the country.	Ministry of Health and Family Welfare, Government of India	Early Childhood Commission & Ministry of Education	National Council for Children’s Services under the Ministry of Education	Ministry of Education	Inter-Ministerial Committee for Early Childhood Development, Department of Social Development
Data Source(s)	https://www.disabilitygateway.gov.au/ads/strategy, https://www.disabilitygateway.gov.au/document/3106, https://www.disabilitygateway.gov.au/sites/default/files/documents/2021-12/1886-tap-early-childhood.pdf	https://www.education.gov.au/early-childhood/about-early-childhood-education-and-care-australia, https://content.legislation.vic.gov.au/sites/default/files/2022-12/10-69aa017-authorised.pdf, https://www.oecd.org/australia/1900259.pdf, https://www.disabilitygateway.gov.au/sites/default/files/documents/2021-11/1786-australias-disability.pdf, https://ourguidelines.ndis.gov.au/early-childhood/early-childhood-approach	https://www.gov.uk/government/publications/early-years-foundation-stage-framework--2, https://www.gov.uk/government/publications/development-matters--2, https://researchbriefings.files.parliament.uk/documents/CDP-2023-0048/CDP-2023-0048.pdf	https://www.gov.uk/government/publications/send-code-of-practice-0-to-25, https://www.gov.uk/government/news/280m-capital-funding-boost-for-children-and-young-people-with-send	https://eclkc.ohs.acf.hhs.gov/, https://www.sciencedirect.com/science/article/pii/S2352827321001919?via%3Dihub	https://sites.ed.gov/idea/, https://sites.ed.gov/idea/, https://sites.ed.gov/idea/files/44th-arc-for-idea.pdf	https://www.acf.hhs.gov/ecd/early-learning/preschool-development-grants, https://www.ffyf.org/issues/pdg/	http://ecd-bangladesh.net/resource/ecd-document-details/10, https://documents1.worldbank.org/curated/en/720311583471084983/pdf/The-Landscape-of-Early-Childhood-Education-in-Bangladesh.pdf	https://www.ncbi.nlm.nih.gov/pmc/articles/PMC10297598/, http://www.planalto.gov.br/ccivil_03/_ato2015-2018/2016/lei/l13257.htm, https://www.planalto.gov.br/ccivil_03/_ato2015-2018/2015/lei/l13146.htm, http://www.scielo.org.co/pdf/rlcs/v5n2/v5n2a02.pdf	https://documents1.worldbank.org/curated/en/992351537159031673/pdf/129940-WP-PUBLIC-Chile-Crece-Contigo-10-a%C3%B1os-FINAL-July-2018.pdf, https://www.bmj.com/content/bmj/363/bmj.k4513.full.pdf, https://www.frontiersin.org/articles/10.3389/fpubh.2022.983513/full, Chile Crece Contigo. Orientaciones Técnicas Salas Inclusivas (2021). Modalidad de Apoyo al Desarrollo Infantil para Niños y Niñas con Discapacidad. Ministerio de Desarrollo Social y Familia, Chile.	https://vikaspedia.in/health/nrhm/national-health-programmes-1/rashtriya-bal-swasthya-karyakram-rbsk#:~:text=The%20services%20aim%20to%20cover,Government%20and%20Government%20aided%20Schools, http://icds-wcd.nic.in	https://www.unicef.org/jamaica/reports/bridging-the-gaps-2019, https://opm.gov.jm/wp-content/uploads/flipbook/jetc-reform-of-education-in-jamaica-2021-abridged/, https://japarliament.gov.jm/attachments/article/339/The-Disabilities-Act-Regulatiojns--2021.pdf	https://planipolis.iiep.unesco.org/sites/default/files/ressources/kenyaecdpolicyframework.pdf, http://guidelines.health.go.ke:8000/media/KENYA_ECD_SERVICE_STANDARD_GUIDELINES__June_2006_FINAL.pdf, http://kenyalaw.org:8181/exist/rest//db/kenyalex/Kenya/Legislation/English/Acts%20and%20Regulations/E/Early%20Childhood%20Education%20Act%20-%20No.%203%20of%202021/docs/EarlyChildhoodEducationAct3of2021.pdf, https://planipolis.iiep.unesco.org/sites/default/files/ressources/kenya_sector_policy_learners_trainees_disabilities.pdf, http://www.parliament.go.ke/sites/default/files/2022-05/REPORT%20ON%20%28%20ECDE%29.pdf	https://platform.who.int/docs/default-source/mca-documents/policy-documents/policy/NGA-CC-10-07-POLICY-eng-Nigeria-IECD-Nat-Policy.pdf, https://planipolis.iiep.unesco.org/sites/default/files/ressources/nigeria_education_sector_reform_bill_draft.pdf, http://wbgfiles.worldbank.org/documents/hdn/ed/saber/supporting_doc/countryreports/ecd/saber_ecd_nigeria_cr_final_2013.pdf	https://www.gov.za/faq/education/what-early-childhood-development

*Although Chile is currently classified as a high-income country by the World Bank, it is still generally regarded as a developing country by the International Monetary Fund (IMF) based on its economic performance.

## 4 Lessons from national ECD programmes

The key findings from the national ECD programmes reviewed are summarised as follows.

### 4.1 ECD is multisectoral and multidisciplinary

Early detection and intervention services for children with disabilities in the first 3 years of life are delivered predominantly within the health sector by diverse professionals including community health workers. The responsibility for the 3–5 years pre-school age is shared by both the health and educational sectors to ensure effective transition to school. Active collaboration between the health and educational sectors with support by the social and finance sectors is recognised as essential to promoting school readiness (29, 30).

### 4.2 Intervention through twin track approach

Many ECD programmes are presented as disability-inclusive, suggesting that all children are provided equal access to all listed services from birth to school entry. However, several cultural, logistical, financial and systemic barriers to equitable access, including discrimination, stereotyping, and stigmatisation are commonly reported (31), making true inclusion for children with disabilities unattainable. Moreover, children with disabilities are not a homogeneous group (6), and many require individualised support to learn and participate on an equal basis with their peers without disabilities (31, 32). Hence, one-size-fits-all ECD programmes often missed or are poorly equipped to serve children with disabilities, particularly those with complex conditions. It also infringes on parental rights and freedom to choose what they consider to be in the child’s best interest.

A twin-track approach has therefore, emerged in several countries like Australia, UK, India and USA, where a disability-inclusive ECD programme is implemented alongside a dedicated disability-focussed ECD programme to optimise access to support services for children with disabilities. Each track is independently managed by a designated department or ministry but coordinated to effectively serve all children with disabilities. For instance, in the USA, disability-inclusive Head Start and Early Head Start programmes under the Department of Health (29), are complemented with disability-focused early intervention programme under the Individuals with Disabilities Education Act (IDEA) coordinated by the Department of Education (30). In the UK, the Early Years Foundation Stage (EYFS) programme for all children under-5 (33), is implemented alongside disability-focused Children with Special Educational Needs and Disabilities (SEND) (34). In India, the Integrated Child Development Services (ICDS) programme which provides early childhood care for all children under-6 (35), is supported with the disability-focused Rashtriya Bal Swasthya Karyakram (RBSK) programme (36).

### 4.3 Guidelines for early detection and intervention

ECD programmes are supported with guidelines for routine newborn screening, developmental screening and surveillance, diagnosis and timely referrals for children with developmental delays and disabilities. Early parenting support is prioritised to ensure family-centred intervention. In some countries, ECD guidelines are integrated with community-based and facility-based maternal and child health services. However, routine newborn screening for developmental disorders and disabilities that is mandated in several HICs is limited in LMICs (37, 38).

### 4.4 Policy supported by disability legislations

National legislations or executive orders are put in place to support ECD programmes for children with disabilities. These laws outline eligibility criteria, service entry points, family involvement, coordinating agencies, performance indicators, workforce training, enforceable rights of children with disabilities and their families, and statutory provisions for funding. Some legislations designate functions to be carried out by various levels of government: national, state and local authorities. Others make explicit provisions for non-state actors including non-governmental organisations. Engagement with and active participation by organisations for people with disabilities (OPDs), adults with lived experience and parent groups is also mandated in line with the UN Conventions of the Rights of the Child (UN-CRC) and the Rights of Persons with Disabilities (UN-CRPD). These legislations facilitate political support for ECD programmes especially for budgetary allocation, and they also provide tools for advocacy.

### 4.5 Establishment of specific funding mechanisms

Funding schemes for implementing service provisions in the ECD legislations are established predominantly in HICs. Most services are federally funded and grants are made to other levels of government based on agreed protocol and responsibilities. The US-based Head Start Programme and Early Intervention and Preschool Education Programme under the IDEA are perhaps the most established and well-funded multi-racial disability-oriented national programmes globally. Funding for IDEA reached $10.8 billion in 2022, supporting states to implement the Act, with $410 million allocated for preschool grants and $496 million for early intervention services. Disability insurance schemes that provide financial assistance directly to families especially where costly support services and assistive technologies are necessary also exist in some countries like Australia. In many LMICs, the absence of federal funding for ECD services contributes to the failure or ineffectiveness of national ECD programmes, even when disability legislations are in place (39, 40).

### 4.6 Monitoring and accountability system

Multisectoral coordination, monitoring and a system of accountability at the national and community levels are provided and legislated in many countries. The accountability mechanisms stipulate roles and responsibilities, rewards and incentives for good performance as well as penalties for poor or non-performance. For example, in the USA, at least 10% of enrolled children must have disabilities and be eligible for special education or early intervention services under the Head Start and Early Head Start programmes. Since inception, these programmes have served over 38 million children and families with up to 13% enrolment of children with disabilities. Before 1975, many disabled children in the USA were excluded from public schools, but in the 2020–21 school year, over 7.5 million received special education services, with more than 66% integrated into general education classrooms under IDEA. Since its inception, India’s RBSK has served approximately 1.2 billion children under 18 years, identifying 86 million with selected impairments or disorders through 360 District Early Intervention Centres.

## 5 Framework for a global disability-focused ECD strategy

Based on the foregoing findings from the national ECD programmes we summarise critical considerations for developing a global disability ECD strategy in this section. It is noteworthy to mention that some of these findings are reinforced by several global ECD reports. For example, a twin-track approach is recommended in the UN Disability Inclusion Strategy (41), the Disability Inclusion Policy and Strategy by UNICEF (42), WHO Disability Policy (43), the World Bank Policy on Disability-Inclusive Health Systems (16), USAID Policy on Inclusive Education (12, 17), and reports from OPDs (31, 44). However, there is no dedicated global ECD strategy for children with disabilities. The NCF already underscores the critical role of a globally coordinated ECD strategy for implementing the global agenda for child development especially in LMICs (19) and has the potential to serve as a pathway for mainstreaming children with disabilities (19). However, because the programme was not originally developed as a disability-inclusive ECD strategy, efforts have been made lately for its adaptation to serve this purpose (45). An independent and complementary disability-focused ECD strategy is now required to ensure targeted support for children with disabilities under the twin-track model.

**Figure 1:**
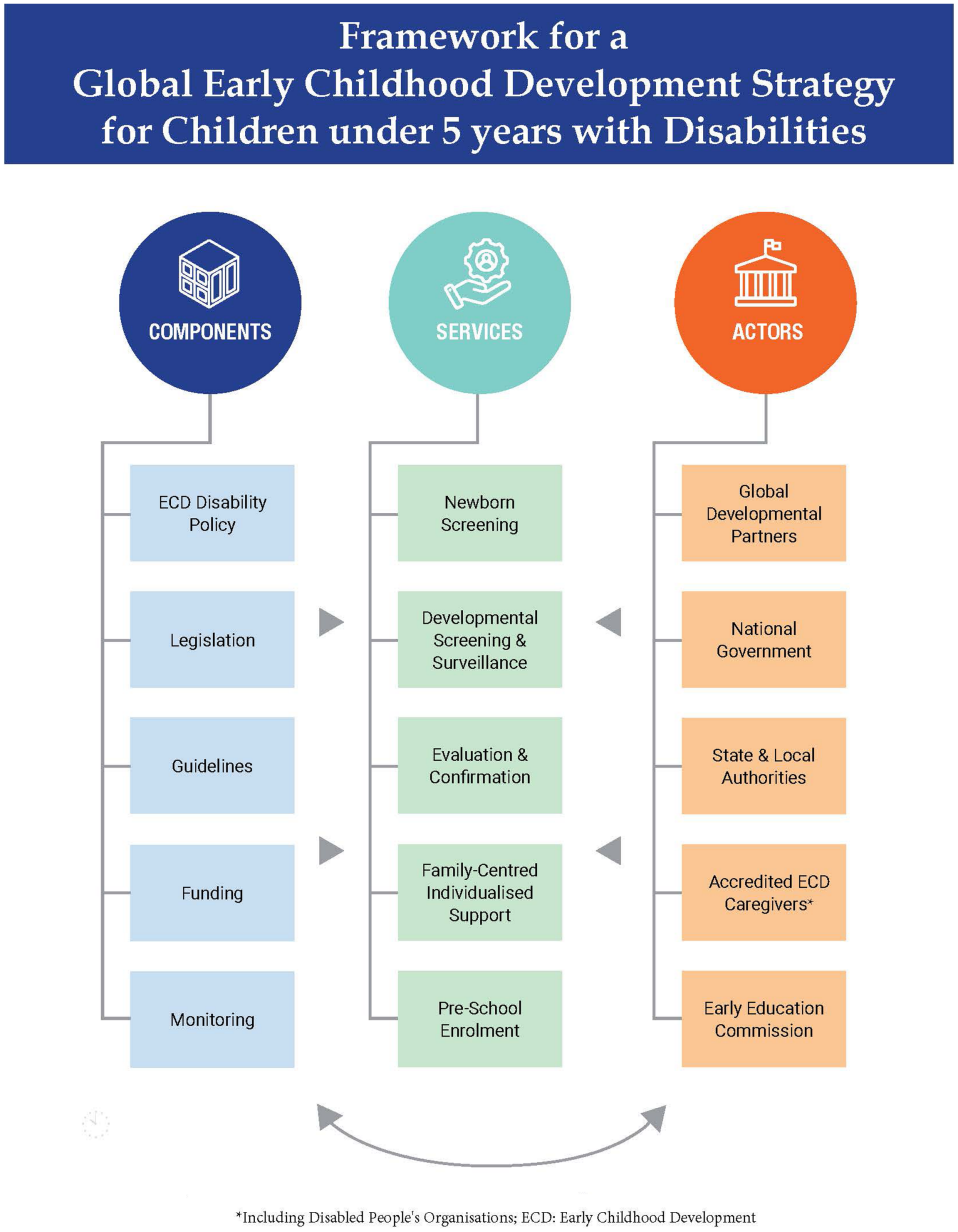
Recommended framework for early childhood development for children under 5 years with disabilities.

A conceptual framework for developing a global disability-focused ECD strategy for children under-5 with disabilities is proposed in Figure 1. The overarching goal and centrepiece of the strategy is to ensure that young children with developmental delays and disabilities are identified early and provided the required support to facilitate equitable access to inclusive education. Within this framework, inclusive education is not merely about integration, merging or mainstreaming. It is the mode of learning that optimises the potential of a child with any degree of disability for inclusion into the wider society. The broad issues to be addressed based on findings from our review are grouped under components of policy issues, services required for timely identification of and intervention for children with disabilities and key actors for implementing the strategy.

### 5.1 ECD policy and services for children with disabilities

A comprehensive global ECD strategy for young children with disabilities ideally should encompass several key components, including a well-defined disability policy, supportive legislation, operational guidelines, sustainable funding mechanism and a robust monitoring system. The proposed policy should address the needs of children with disabilities from birth up to age 5 years, emphasising the critical role of early intervention from birth as a foundation for pre-school education. Early intervention before age 2–3 years has been demonstrated in longitudinal studies to reduce the need for special education for some children with developmental delays and disabilities at school entry (46, 47). The exclusion of children under 2 years from the current indicator for SDG 4.2 and the UNICEF monitoring tool (ECDI2030) therefore, needs to be resolved as soon as possible (48). The ECDI2030 should be harmonised with the Global Scale for Early Development for children aged 0–3 years recently developed by WHO to produce a single tool for children under-5 for use in all LMICs. The global disability policy should make explicit provisions for the required health and educational services including newborn and developmental screening and surveillance ideally synchronised with well-child visits including routine immunisation, referral pathway for diagnostic evaluation, and links for timely enrolment into family-centred support programmes for children with disabilities (49, 50). The absence of routine newborn screening for developmental disorders and disabilities in the vast majority of many LMICs needs to be addressed to ensure the timely detection of children with disabilities. Early parenting interventions should be emphasised to minimise the emotional burden and sense of helplessness often encountered by parents following diagnosis of disability in their child (51, 52). The policy should also provide clear guidance on how to manage the transition from family-based intervention services in the first 3 years of life to pre-school enrolment starting at age 3 years.

The global ECD policy needs to be supported by appropriate legislations (4, 53–55). All countries included in our review had specific disability laws or general legislations for all children with or without reference to children with disabilities. The UN Conventions of the Rights of the Child and the Rights of Persons with Disabilities already provide a practical framework and guidance for mandating all countries to make specific provisions for children under-5 in their national laws for persons with disabilities and childcare legislations. A template can be developed to assist countries with and without disability legislations to make specific legally binding provisions for services required by children with disabilities in line with SDG 4.2. Such legislations also empower parents to seek their rights to state support for their children.

Comprehensive operational guidelines for service providers across all levels of service delivery are necessary. These guidelines should be adaptable to different populations and should reflect the standard protocol for clinical recommendations and guidelines provided by WHO. The guidelines should address the range of services listed in the framework as a matter of principle and best practice. Even in situations where ideal technologies may not be readily available, these guidelines can serve as a valuable compass, informing service providers about the desired direction and potential avenues for future improvement.

Disability policies and legislations are necessary but not sufficient without funding. The critical role of funding is reinforced in various global reports on disability inclusion (Appendix 1). Many LMICs rely on funding from donor organisations and HICs for maternal and child health programmes (56) and are more likely to require such support for ECD initiatives (57). In our view, the introduction of a global disability-focussed ECD strategy is likely to attract greater attention and funding. At the current levels of developmental assistance to LMICs for childhood disabilities by OECD donors and others, the global ECD agenda is unachievable (58). A dedicated global fund should be considered to support LMICs that have instituted appropriate legislations for children with disabilities and are committed to allocating a proportion of their annual health and educational budgets for ECD services.

Global funding schemes require effective monitoring system for accountability linked to specific performance indicators (16, 41–43, 55). For example, it is important to track the number of children that are screened, identified with disabilities, enrolled for early intervention services, and attending preschool programmes during each reporting period. Ongoing access to global funds should be contingent on these performance indicators.

### 5.2 Key actors and way forward

The key actors with critical roles in ensuring the effectiveness of the global ECD Strategy include donor organisations, relevant government ministries, state and local authorities, accredited providers of services for children with disabilities at community-level, including OPDs and a designated national governance body. Countries may consider establishing an independent but multidisciplinary ECD Commission with specific mandate for inclusive education in line with all the provisions of the SDG 4.2. In line with the efforts to transform the NCF into a global disability-inclusive ECD programme, it will be necessary to designate lead UN agencies for the development and implementation of the global disability-focussed ECD strategy. Additionally, we recommend that in developing a comprehensive global strategy as proposed in this paper, the relevant UN agencies should consider engaging with administrators of established national ECD programmes in different world regions for better insights on the associated operational challenges and how to address them. Lessons and key performance indicators (e.g., school enrolment and participation, school completion rate, programme costs) that have not been published can be garnered from such engagements to inform the introduction of global and national targets. Also, it is important to clarify that the implementation of a global strategy is typically country-led, allowing nations to adapt and prioritise service delivery within a defined operational framework to promote a greater sense of ownership and best possible developmental outcomes across diverse cultures and contexts. We are not unmindful of several cultural, health and social barriers to service delivery and uptake that persist even in countries with well-established and well-funded ECD programmes especially in high-income countries (59–62). A global disability-focussed ECD strategy is unlikely to fully address the stigma and discrimination faced by children with disabilities and their families worldwide as they transition into school education in inclusive settings. However, it provides a pathway for individualised support especially for children with severe or complex disabilities.

## 6 Conclusion

The global ECD commitment under the SDGs requires that disabled children and their families are empowered from birth for equitable access and participation in the larger society through inclusive and quality education. This aspiration is supported by disability-inclusion policies of various UN agencies and OPDs since 2015. Evidence from well-established ECD national programmes have shown that children with disabilities and their families are better served through a twin track approach in which a dedicated and disability-focused ECD strategy is implemented alongside disability-inclusive ECD programmes for all young children. This review provides a framework for developing an independent global disability-focussed ECD strategy aimed at ensuring that children with disabilities and their families are adequately served in all countries. It is unlikely that the vision and commitment under the SDGs for children with disabilities will be realised without such a strategy under a dedicated global leadership.

## Author contributions

BO: Writing – review & editing, Writing – original draft, Visualization, Validation, Supervision, Software, Resources, Project administration, Methodology, Investigation, Funding acquisition, Formal analysis, Data curation, Conceptualization. SW: Writing – review & editing, Supervision. TS: Writing – review & editing, Writing – original draft, Supervision, Data curation. MK: Writing – review & editing, Writing – original draft, Data curation. MM-A: Writing – review & editing, Data curation. SG: Writing – review & editing. SB: Writing – review & editing, Data curation. NA: Writing – review & editing. MF: Writing – review & editing. LG: Writing – review & editing. OO: Writing – review & editing. PL: Writing – review & editing. BB: Writing – review & editing. AW: Writing – review & editing. JO: Writing – review & editing, Supervision, Data curation. DW: Writing – review & editing. AD: Writing – review & editing, Supervision. MH-A: Writing – review & editing, Supervision. MG: Writing – review & editing, Writing – original draft, Supervision. The authors are members of the Global Research on Developmental Disabilities Collaborators (GRDDC) - a diversified group of caregivers with and without lived experience of disability from various socio-cultural and income settings, along with parents of children with disabilities.
