# Supplementary material for: Early childhood development strategy for the world’s children with disabilities
Source: Front Public Health. 2024 Jun 19;12:1390107. doi: 10.3389/fpubh.2024.1390107 (PMC11220280; doi:10.3389/fpubh.2024.1390107)
Supplement: Supplementary file 1 [file Presentation_1.pdf]

Appendix 1. Global reports reviewed on early childhood development or disability inclusion for children with disabilities

Organisation	Year	Report Title [Link to source accessed on 20 February 2024]
**United Nations**	2015	Sustainable Development Goals (SDGs). http://www.un.org/sustainabledevelopment/sustainable-development-goals
	2019	United Nations Disability Inclusion Strategy. https://www.un.org/en/content/disabilitystrategy/
**UNICEF**	2023	Progress on Children's Well-Being: Centring child rights in the 2030 Agenda; For every child, a sustainable future. https://data.unicef.org/resources/sdg-report-2023/
	2023	Early Childhood Development. UNICEF Vision for Every Child. https://www.unicef.org/media/145336/file/Early%20Childhood%20Development%20-%20UNICEF%20Vision%20for%20Every%20Child.pdf
	2023	Mapping and Recommendations on Disability-Inclusive Education in Eastern and Southern Africa. https://www.unicef.org/esa/media/12201/file/Full_Report_Mapping_of_Progress_towards_disability-inclusive_in_ESA.pdf
	2022	Disability Inclusion Policy and Strategy (DIPAS) 2022–2030. https://www.unicef.org/unicef-disability-inclusion-policy-and-strategy-dipas-2022-2030
	2022	Inclusion Matters: Inclusive Interventions for Children with Disabilities – An evidence and gap map from low- and middle-income countries. https://www.unicef-irc.org/publications/1537-inclusive-interventions-for-children-with-disabilities-in-low-and-middle-income-countries-an-evidence-gap-map.html
	2021	Seen, Counted, Included: Using data to shed light on the well-being of children with disabilities. https://data.unicef.org/resources/children-with-disabilities-report-2021/
**WHO**	2022	Global report on health equity for persons with disabilities. https://www.who.int/publications/i/item/9789240063600
	2022	Optimizing brain health across the life course: WHO position paper. https://iris.who.int/bitstream/handle/10665/361251/9789240054561-eng.pdf?sequence=1
	2021	WHO policy on disability. https://iris.who.int/bitstream/handle/10665/341079/9789240020627-eng.pdf?sequence=1
	2019	Rehabilitation in health systems: guide for action. https://iris.who.int/bitstream/handle/10665/325607/9789241515986-eng.pdf?sequence=1

**UNICEF & WHO**	2023	Global report on children with developmental disabilities: from the margins to the mainstream. https://www.unicef.org/documents/global-report-children-developmental-disabilities
	2023	Nurturing care framework progress report 2018-2023: reflections and looking forward. https://iris.who.int/bitstream/handle/10665/369449/9789240074460-eng.pdf?sequence=1
	2022	Nurturing Care Handbook: operationalising the Nurturing Care Framework. https://nurturing-care.org/handbook/
	2022	Nurturing care practice guide: strengthening nurturing care through health and nutrition services. https://iris.who.int/bitstream/handle/10665/365601/9789240058651-eng.pdf?isAllowed=y&sequence=1
	2018	Nurturing care for early childhood development: a framework for helping children survive and thrive to transform health and human potential. http://apps.who.int/iris/bitstream/handle/10665/272603/9789241514064-eng.pdf?ua=1
**The World Bank**	2023	Approaches to Deliver Inclusive Education in Sub-Saharan Africa and South Asia. https://openknowledge.worldbank.org/server/api/core/bitstreams/ba849b58-e43a-4976-932c-320649f36451/content
	2022	Disability-Inclusive Health Care Systems: Technical Note for World Bank Task Teams. https://openknowledge.worldbank.org/server/api/core/bitstreams/6a009b21-c3e0-50aa-aa7c-6677a1dedf2e/content
	2020	Pivoting to Inclusion: Leveraging Lessons from the COVID-19 Crisis for Learners with Disabilities. https://documents1.worldbank.org/curated/en/777641595915675088/pdf/Pivoting-to-Inclusion-Leveraging-Lessons-from-the-COVID-19-Crisis-for-Learners-with-Disabilities.pdf
	2019	The Inclusive Education Initiative (IEI) https://www.inclusive-education-initiative.org/index.php/knowledge-repository
**UNESCO**	2023	The 2023 Global Education Monitoring (GEM) Report. https://unesdoc.unesco.org/ark:/48223/pf0000385723
	2021	Inclusion in early childhood care and education : Brief on inclusion in education. https://unesdoc.unesco.org/ark:/48223/pf0000379502
	2021	Welcoming learners with disabilities in quality learning environments: a tool to support countries in moving towards inclusive education https://unesdoc.unesco.org/ark:/48223/pf0000380256/PDF/380256eng.pdf.multi

	2020	Global education monitoring report, 2020, Latin America and the Caribbean: inclusion and education: all means all. https://unesdoc.unesco.org/ark:/48223/pf0000374614
**The United States Agency for International Development (USAID)**	2023	Global Child Thrive Act: Implementation Guidance. https://www.advancingnutrition.org/resources/global-thrive-act-implementation-guidance
	2020	"Are we fulfilling our promises? Inclusive education in Sub-Saharan Africa?" https://www.edu-links.org/resources/are-we-fulfilling-our-promises-inclusive-education-sub-saharan-africa
	2018	How-To Note: Disability Inclusive Education. https://inee.org/sites/default/files/resources/How-ToNote_DisabilityInclusiveEducation_0.pdf
**Department for International Development (DFID)**	2018	Diversity and inclusion strategy 2018 to 2025. https://assets.publishing.service.gov.uk/media/5b9787c1e5274a139df4d439/Diversity_and_Inclusion_strategy_SCREEN.pdf
**OECD**	2017	Key OECD Indicators on Early Childhood Education and Care, Starting Strong. https://www.oecd.org/education/starting-strong-2017-9789264276116-en.htm
**The International Disability and Development Consortium (IDDC)**	2023	Inclusive Education: an imperative for advancing human rights and sustainable development. https://www.iddcconsortium.net/blog/inclusive-education-an-imperative-for-advancing-human-rights-and-sustainable-development/
**International Disability Alliance (IDA)**	2020	What an Inclusive, Equitable, Quality Education means to us. https://www.internationaldisabilityalliance.org/sites/default/files/ida_ie_flagship_report_english_29.06.2020.pdf
**The Global Action on Disability Network (GLAD)**	2021	GLAD Network's 2021-2023 Strategic Plan. https://gladnetwork.net/sites/default/files/2021-03/2021-2023%20GLAD%20Strategic%20Plan.pdf
**Christoffel-Blindenmission (CBM) Global**	2017	Disability Inclusive Development Toolkit https://cbm-global.org/wp-content/uploads/2022/05/CBM-DID-TOOLKIT-accessible.pdf
**Early Childhood Development Task Force (ECDtf)**	2019	Global Survey of Inclusive Early Childhood Development and Early Childhood Intervention Programs. https://www.unicef.org/media/126046/file/Global-Survey-of-IECD-and-ECI-Programs-2019.pdf
